# Feasibility of intravitreal injections and ophthalmic safety assessment in marmoset (*Callithrix jacchus*) monkeys

**DOI:** 10.5194/pb-4-93-2017

**Published:** 2017-04-28

**Authors:** Birgit Korbmacher, Jenny Atorf, Stephanie Fridrichs-Gromoll, Marilyn Hill, Sven Korte, Jan Kremers, Keith Mansfield, Lars Mecklenburg, Andrew Pilling, Andreas Wiederhold

**Affiliations:** 1 Covance Preclinical Services GmbH, Kesselfeld 29, 48163 Münster, Germany; 2 Department of Ophthalmology, University Hospital Erlangen, Maximiliansplatz 2, 91054 Erlangen, Germany; 3 Novartis Pharma AG, Basel, Klybeckstraße, 4002 Basel, Switzerland

## Abstract

To safeguard patients, regulatory authorities require that new drugs that are to be given by the intravitreal (IVT) route are assessed for their safety in a laboratory species using the same route of administration. Due to the high similarity of ocular morphology and physiology between humans and nonhuman primates (NHPs) and due to the species specificity of many biotherapeutics, the monkey is often the only appropriate model. To this end, intravitreal administration and assessment of ocular toxicity are well established in cynomolgus monkeys (*Macaca fascicularis*). In contrast, the common marmoset monkey (*Callithrix jacchus*) is not a standard model for ocular toxicity studies due to its general sensitivity to laboratory investigations and small eye size. It was the purpose of the present work to study whether the marmoset is a useful alternative to the cynomolgus monkey for use in intravitreal toxicological studies. Six marmoset monkeys received repeated (every 2 weeks for a total of four doses) intravitreal injections of 10 or 20 µL of a placebo. The animals were assessed for measurements of intraocular pressure (IOP), standard ophthalmological investigations and electroretinography (ERG). At the end of the dosing period, the animals were sacrificed and the eyes were evaluated histologically. ERG revealed similar results when comparing predose to end-of-study data, and there was no difference between the two dose volumes. A transient increase in the IOP was seen immediately after dosing, which was more pronounced after dosing of 20 µL compared to 10 µL. Ophthalmologic and microscopic observations did not show any significant changes. Therefore, it can be concluded that 10 µL as well as 20 µL intravitreal injections of a placebo are well tolerated in the marmoset. These results demonstrate that the common marmoset is an alternative to the cynomolgus monkey for intravitreal toxicity testing.

## 1 Introduction

**Figure 1:**
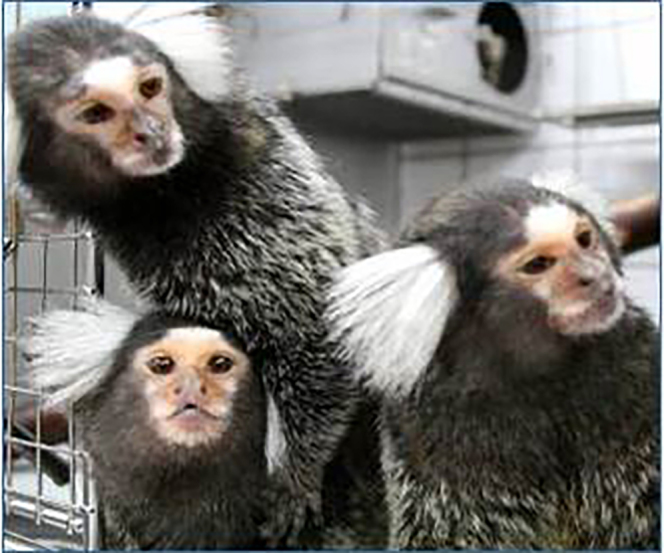
Common marmoset monkey (*Callithrix jacchus*).

To support clinical trials of new drugs to be given by the intravitreal (IVT) route, it is required to assess the safety after IVT administration to a laboratory species. Due to the high similarity of ocular morphology and physiology between humans and nonhuman primates (NHPs) and given the species specificity of many biotherapeutics, the monkey often is the only appropriate species for preclinical safety testing. To this end, intravitreal administration procedures and assessments of ocular toxicity in cynomolgus monkeys (*Macaca fascicularis*) are well established (Niggemann et al., 2006). In contrast, the common marmoset monkey (*Callithrix jacchus*; Fig. 1) is not a standard model for ocular toxicity studies due to its general sensitivity to laboratory investigations and small eye size.

However, apart from a polymorphism in red–green color vision (Mollon et al., 1984 and Travis et al., 1988), only minor structural and functional differences in the retina were described in comparison to macaques. In addition, recordings from single neurons in the lateral geniculate nucleus showed that the functional properties of visually responsive cells in marmosets are very similar to those in the macaque (Kremers et al., 1999). It was the purpose of the present work to study whether the marmoset is a useful alternative to the cynomolgus monkey for use in intravitreal toxicological studies and whether injecting a volume of 10 or even 20 µL would be tolerated.

## 2 Materials and methods

### 2.1 Pre-study test

To determine various weight and anatomical measurements, seven eyes from four adult common marmosets, that were sacrificed for reasons unrelated to this study, were obtained at necropsy, and following removal of periorbital muscle and fat, the eyes were snap-frozen at -70 ${}^{\circ }$C. Eyes were thawed and weighed. Transverse and sagittal measurements were performed. Volumetric measurements were made by recording fluid displacement in a 10.0 mL graduated cylinder. The eye was then dissected and similar measurements were recorded for the vitreous and lens.

### 2.2 Housing, ethical and regulatory guideline considerations

During the development of a specific biotechnology-derived compound, it was shown that the marmoset was the only pharmacologically relevant species for nonclinical safety testing. Due to the very limited experience with IVT administration in marmosets (Ivanova et al., 2010; Melo et al., 2012; Neitz et al., 2013), this feasibility study was conducted in order to prepare for a good laboratory practice (GLP) toxicity study with this specific compound.

The test facility Covance Preclinical Services GmbH (Germany) is fully accredited by AAALAC International. All procedures in this study were in compliance with the German Animal Welfare Act and were approved by the local Institutional Animal Care and Use Committee (IACUC). Furthermore, the procedures were performed in consideration of the Directive 2010/63/EU of the European Parliament and of the Council of 22 September 2010 on the protection of animals used for scientific purposes.

The standard social housing for marmosets at Covance, Münster (Germany), is according to the “Commission Recommendation 2007/526/EC on guidelines for the accommodation and care of animals used for experimental and other scientific purposes (Appendix A of Convention ETS 123)”. The standard cage allows three-dimensional movements by this highly agile primate species in groups of two or three individuals.

The animals received a variety of food, which was prepared freshly each day and given twice a day according to a meal plan. The room temperature was between 22 and 28 ${}^{\circ }$C, with a relative humidity between 40 and 70 %, and artificial lighting was in 12 h dark/light cycle. The cages were enriched with sleeping boxes, wooden chips, plastic balls and wooden bars.

### 2.3 Feasibility study

The animals were 1 to 10 years old and weighed between 349 and 501 g. One group of three male marmoset monkeys received repeated bilateral intravitreal injections of 10 µL of a placebo (70 mM mannitol, 20 mM histidine pH 6.5, and 0.04 % polysorbate) every second week (days 1, 15, 29, and 43). The second group of three male animals received 20 µL of a placebo on the same days. Ophthalmic examinations and intraocular pressure (IOP) measurements were performed once before there start of dosing (predose), on days 1 (directly after dosing), 3, 15 (directly after dosing), 17, 29 (directly after dosing), 31, 43 (directly after dosing) and 45 (before the animals were sacrificed as scheduled). Electroretinography (ERG) was performed twice before the start of dosing and at week 6 of the study (after the last administration of the placebo).

#### 2.3.1 Intravitreal dosing

**Figure 2:**
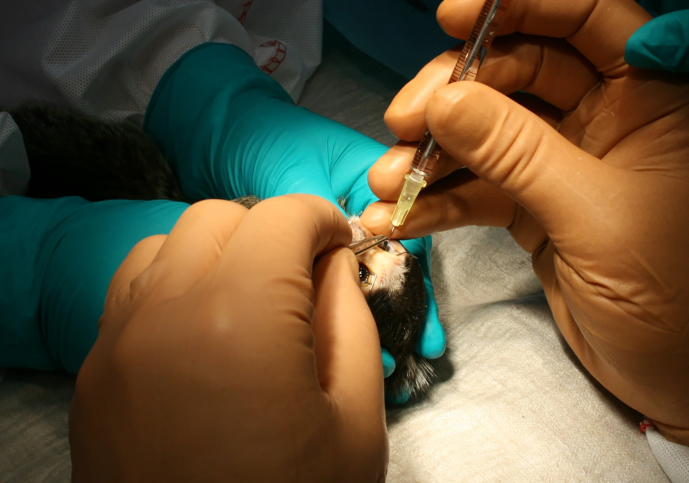
Intravitreal administration in the Marmoset eye.

The animals were fasted before they were anesthetized by intramuscular injection of ketamine and medetomidine. Mydriasis was induced by using 1 % tropicamide eye drops, and an antiseptic (povidone-iodine 5 %) solution was instilled in the area of the injection. A local ophthalmic anesthetic was instilled into both eyes prior to insertion of a lid speculum. A microscope was used for dose administration with the aim to observe any reflux (see Fig. 2). The distance from the corneal limbus was measured (approx. 1.5 to 2.0 mm) and marked on the conjunctiva. The needle was inserted approximately 3 mm into the vitreous body in the direction to the posterior pole. The needle stayed in that position for 3–5 s after dosing so that the vehicle could distribute in the vitreous body. Before the needle was removed it was clasped with a Colibri forceps to avoid reflux. Reflux could not be completely avoided but was only seen on few occasions.

Ointment containing dexpanthenol was instilled onto each eye following the dosing, and atipamezole was used as an anesthetic antidote at the end of the procedure (intramuscular injection).

#### 2.3.2 Ophthalmic examinations

Ophthalmic examinations were conducted following sedation using ketamine. Mydriasis was induced by using 1 % tropicamide eye drops. The eye and the connecting tissues were evaluated during the macroscopic investigations. The ocular fundus with the macula lutea, papilla, ocular vessels and retina was assessed funduscopically. Slit lamp examinations including fluorescein staining were performed to evaluate the anterior and medium segment of the eye with conjunctiva, cornea, anterior chamber, iris, lens and vitreous body. In addition, the intraocular pressure was determined using the TonoVet^®^ tonometer.

#### 2.3.3 Electroretinography

The animals were dark adapted for at least 30 min prior to anesthesia using ketamine and medetomidine. The pupils were dilated with 1 % tropicamide eye drops and 0.5 % atropine eye drops. Further preparation of the animals was performed under dimmed red light to ensure that rod sensitivity was preserved.

For the first measurement, ERGs were only measured in the right eyes using DTL (Dawson–Trick–Litzkow) electrodes that were placed over the lower conjunctiva of each eye, serving as active electrodes. The eyes were covered by methylcellulose and a custom-made contact lens (radius of curvature: 3.5 mm; 5 mm diameter; 0 diopter; Cantor & Nissel, UK) to avoid desiccation of the eye. During the second measurement and the dosing phase, the left and right eyes were measured simultaneously and the DTL electrodes and contact lenses were replaced by contact lens electrodes (same properties as above; Mayo Corporation, Japan). The contact lens electrodes were easier to handle and gave nearly identical results as the DTL electrodes. The data obtained with both techniques were considered comparable. Needle electrodes that were placed subcutaneously at the base of the ipsilateral ears served as references. Ground needle electrodes were placed at the base of the tail. The animals were placed on a platform that could be slid into the full-field stimulator. Each recording lasted approximately 30 min.

**Table 1:** Ocular weight and linear and volumetric measurements from adult common marmoset eyes.

	Animal 1	Animal 2	Animal 2	Animal 3	Animal 3	Animal 4	Animal 4	Mean	SD
	Eye 1	Eye 1	Eye 2	Eye 1	Eye 2	Eye 1	Eye 2		
Eye weight (g)	0.59	0.749	0.7412	0.6489	0.6618	0.7472	0.7366	0.70	0.058
Lens weight (g)	0.019	0.027	0.026	0.030	0.041	0.026	0.051	0.03	0.010
Lens wt / eye wt	0.03	0.04	0.03	0.03	0.07	0.03	0.07	0.04	0.017
Circumference (A)	3.3	3.8	3.8	3.4	3.4	3.9	3.9	3.6	0.244
Transverse (cm)									
Circumference (B)	3.5	3.9	3.9	3.6	3.5	3.8	3.8	3.7	0.164
Sagittal (cm)									
Lens volume (mL)	n/a	0.03	0.03	0.03	0.04	0.01	0.025	0.03	0.009
Vitreous weight (g)	n/a	0.558	0.552	0.424	0.487	0.483	0.587	0.52	0.056
Vitreal wt / total eye wt	n/a	0.74	0.74	0.65	0.8	0.65	0.80	0.73	0.062
Vitreous volume (mL)	0.4	0.6	0.6	0.6	0.6	0.5	0.5	0.54	0.073
Total volume (mL)	0.6	0.8	0.8	0.8	0.8	0.7	0.8	0.76	0.073
Vitreal vol / total vol	0.67	0.75	0.75	0.71	0.63	0.71	0.63	0.69	0.049

The animals underwent ERG measurements to the following five stimulus protocols:

1. Scotopic flash ERGs were recorded with a dark background. The responses to six flash strengths (0.0095, 0.03, 0.095, 0.3, 0.95 and 3.0 cd⋅s m${}^{-2}$) were recorded. The flash frequency was 0.3 Hz. The recordings to six flashes were averaged. The a-waves were measured only at the highest flash strength (i.e., 3.0 cd m${}^{-2}$) from baseline to the trough of the a-wave. The b-waves were measured at all flash strengths. The amplitudes were defined as the voltage difference between the trough of the a-wave to the peak of the b-wave.
2. Oscillatory potentials (OPs) were measured for four flashes on a dark background at a rate of 0.1 Hz. The flash strength was 3.0 cd⋅s m${}^{-2}$. Representative for all OPs, the OP amplitude between the peak of OP2 and the following trough was measured. The latency was defined as the time to the trough following OP2.
3. Photopic flicker ERGs were recorded for a train of 3.0 cd⋅s m${}^{-2}$ flashes on a 100 cd m${}^{-2}$ background at a rate of 30.1 Hz. The responses to 50 flashes were measured and averaged. The measurements were performed directly after the background was switched on and were repeated after 10 min of adaptation. A general increase in the ERG responses was observed during this time. Therefore, only the second measurement was used for further analysis. The response amplitude was defined as a trough-to-peak amplitude.
4. The responses to red flashes on a 100 cd m${}^{-2}$ white background were recorded. Two flash strengths were used: 0.3 and 0.95 cd⋅s m${}^{-2}$. The flash rate was 1.5 Hz and the responses to 30 flashes were averaged. White backgrounds were chosen instead of blue backgrounds to be able to compare the data with previous data obtained from cynomolgus monkeys. The white backgrounds were photopic and thus sufficiently suppressed rod-driven signals. The b-wave amplitudes and implicit times were measured.
5. The photopic flashes were repeated with white flashes. The responses to 20 flashes of 3.0 cd⋅s m${}^{-2}$ strength were averaged. The flash rate was 1.5 Hz. The a- and b-wave amplitudes and latencies were measured.

#### 2.3.4 Necropsy and histopathology of the eye

**Figure 3:**
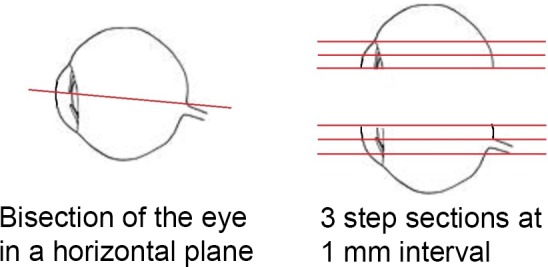
Trimming and sectioning of the eye.

Animals were sedated by intramuscular injection of ketamine followed by an intravenous sodium pentobarbitone overdose prior to exsanguination. Both eyes including the optic nerve were fixed in Davidson's fluid. The eye was bisected in a horizontal plane, just below the equator, and embedded in paraffin wax. Sections were prepared at a nominal thickness of 5 µm. Three serial sections were taken through each block at 1 mm spacing, yielding a total of six sagittal sections per eye (Fig. 3). These sections included the optic disc and the fovea. Sections were stained with hematoxylin and eosin (H&E).

## 3 Results

### 3.1 Pre-study test

Weights and linear and volumetric measurements are provided in Table 1. Total mean vitreal volume was determined at 0.54 mL and mean vitreal weight at 0.52 g. The mean vitreal wt / total eye weight ratio was 0.73 and is comparable to the ratio of 0.65 reported for cynomolgus monkey eyes using a frozen dissection method (Struble et al., 2014).

### 3.2 Feasibility study

#### 3.2.1 ERGs

**Figure 4:**
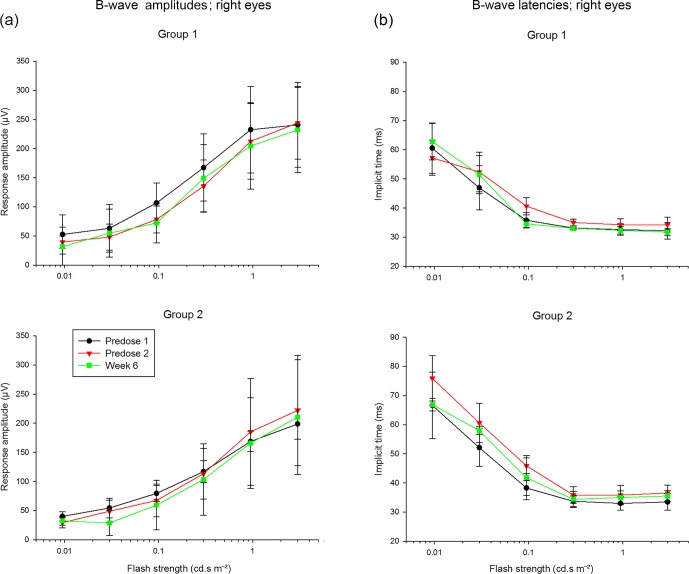
**(a)** Scotopic b-wave amplitudes. **(b)** Scotopic b-wave latencies.

Representative for all ERG parameters, Fig. 4a and b show the b-wave amplitudes and latencies of the scotopic electroretinograms, respectively, of the right eyes of both treated groups measured twice before injections (predose 1 and 2) and 6 weeks after injections. The ERG assessment revealed similar results when comparing predose to end-of-study data, and there was no difference between the two dose volumes.

**Table 2:** ERG reference data from 46 cynomolgus monkeys (right eye) in comparison to the mean of the three marmosets (right eye) from group 1 before dosing.

Standard response	Species	b-wave	b-wave peak	a-wave	a-wave peak	Flash	Adaptation
		amplitude	latency	amplitude	latency	intensity	status
		(µV)	(ms)	(µV)	(ms)	(mcds m${}^{-2})$	
rod response	cyno	119 [29]	50 [4]	–	–	95	dark
	marmoset	78 [23]	41 [3]				(30 min)
maximal response	cyno	194 [41]	38 [2]	95 [24]	17 [2]	3000	dark
	marmoset	244 [62]	34 [3]	123 [9]	15 [0.4]		
oscillatory potential	cyno	18 [6]	25 [1]	–	–	3000	dark
	marmoset	16 [11]	24 [1]	–	–		
30 Hz flicker	cyno	77 [20]	57 [0.9]	–	–	3000	light
	marmoset	145 [5]	58 [0.5]	–	–		(10 min)
red flash cone response	cyno	41 [13]	24 [2]	–	–	950	light
	marmoset	116 [5]	22 [0.5]				
white flash cone response	cyno	88 [25]	24 [0.8]	16 [3]	14 [0.9]	3000	light
	marmoset	177 [17]	26 [0.7]	48 [10]	15 [0.4]		

[ ] = standard deviation.

**Figure 5:**
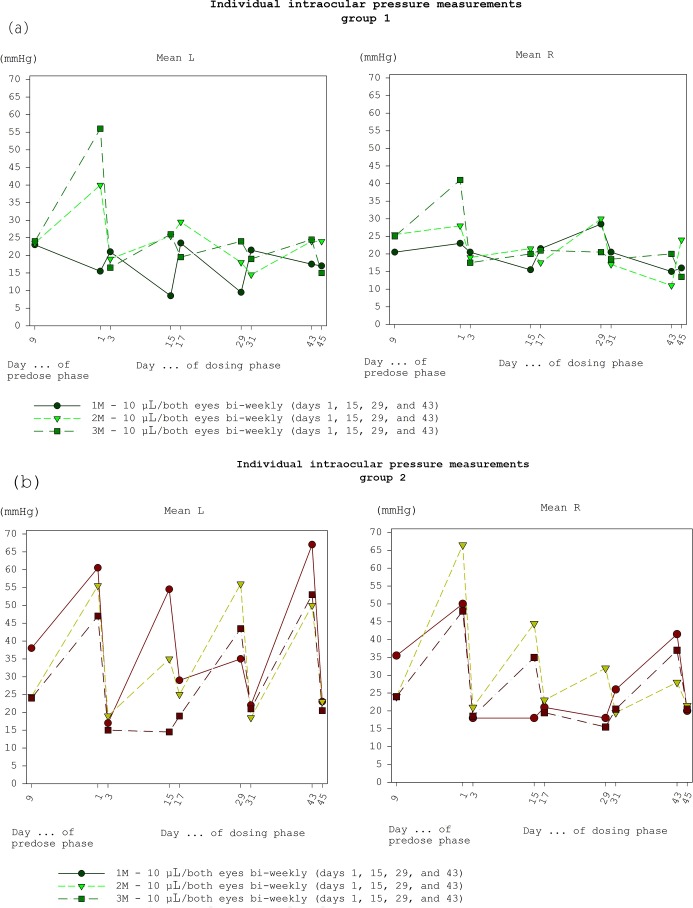
**(a)** Intraocular pressure of group 1 (10 µL eye${}^{-1}$). **(b)** Intraocular pressure of group 2 (20 µL eye${}^{-1}$).

None of the measured parameters showed any difference between data obtained from the left and right eyes. The variability in the data was satisfactory (see Fig. 4). We averaged all amplitude and latency data for each stimulus condition (i.e., obtained from the two eyes, from the three experimental sessions and from all animals in the two groups). The scotopic b-wave amplitudes deviated from the averages up to about 30 % from these averages for low-intensity flashes. At high intensities the deviation was maximally 15 %. The deviation in all other parameters was generally less than 10 %, except for the OP amplitudes and the b-wave amplitude obtained with the dimmer (0.3 cd⋅s m${}^{-2}$) red flash, where the deviations could be larger probably due to the smaller amplitudes and thus less favorable signal-to-noise ratio (similar to the scotopic b-wave amplitude at low flash strengths).

In agreement with macaque and human data, the amplitudes increase with increasing flash strength. Furthermore, the implicit times decrease with increasing stimulus strength. The absolute values of the scotopic a- and b-wave amplitudes measured with 3 cd⋅s m${}^{-2}$ flashes agree with those measured in humans at the same conditions (Hamilton et al., 2015). The implicit time of the a-wave at the same flash strength was also similar to those measured in human subjects. The latency of the b-wave was, however, shorter (35 ms) than those measured by Hamilton et al. (2015) in human subjects (about 50 ms).

In general, the responses to the different flash intensities are comparable to those measured in cynomolgus monkeys (Table 2). However, the cone-driven response appears to be stronger than in cynomolgus monkeys. This is likely to be related to the higher cone density found in marmosets in comparison with macaques (Goodchild et al., 1996).

#### 3.2.2 Ophthalmic examinations

Slit lamp examination and funduscopy did not show any changes in ocular structures which could be due to manipulation.

#### 3.2.3 Intraocular pressure

A transient increase in the intraocular pressure was seen immediately after dosing which was more pronounced after dosing of 20 µL compared to 10 µL (see Fig. 5a and b) indicating the influence of the volume to the intraocular pressure immediately following dosing. Pressure increase indicates that 20 µL is about the maximum volume injectable without damage to the intraocular structures.

#### 3.2.4 Histology

**Figure 6:**
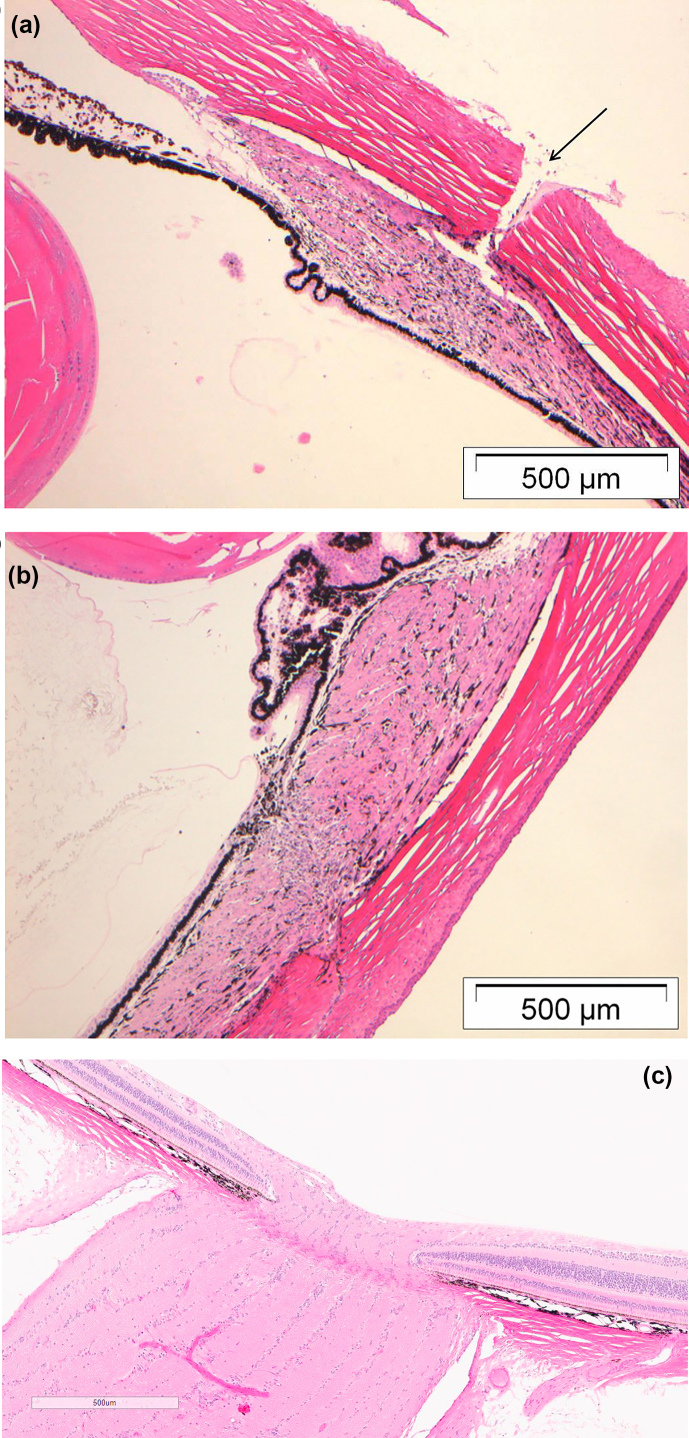
**(a)** H&E-stained section from the eye of a marmoset monkey treated intravitreally (10 µL). The image shows the ciliary body and portions of the iris, sclera and lens. Note the needle track lesion in the sclera (arrow) and the disorganization of collagen fibers in the pars plana of the ciliary body, accompanied by few inflammatory cell infiltrates. **(b)** H&E-stained section from the eye of a marmoset monkey treated intravitreally (10 µL). This picture shows the disorganization of the stromal layers and minimal inflammatory cell infiltrates. **(c)** H&E-stained section from the eye of a marmoset monkey treated intravitreally (20 µL). This picture shows the normally structured papilla of the optic nerve with no microscopical changes induced by transiently increased intraocular pressure.

Microscopic findings were limited to the site of injection (Fig. 6a) and were characterized by minimal focal disorganization of stromal layers mainly affecting the muscle of the ciliary body (Fig. 6b) and a subepithelial infiltrate of inflammatory cells of the conjunctiva at the corneoscleral limbus. The cell infiltrate was composed of neutrophils and/or mononuclear cells, was minimal in magnitude, and was focal or multifocal in distribution. Disorganization of stromal layers and inflammatory cell infiltrates occurred to the same extent in animals from the 10 and the 20 µL dose volume group. There was no difference in magnitude between the groups, no morphological evidence of occlusion of the iridocorneal angle, and no evidence of optic nerve or retinal damage from increased intraocular pressure (Fig. 6c).

## 4 Discussion

During pretest evaluation of marmoset eyes, the volume of the vitreous body was determined at 0.54 mL. In cynomolgus monkeys the volume was determined at 2 mL (Struble at al., 2014) using the same technique. Since the standard injection volume in cynomolgus monkey is 50 µL per eye, the volume of 10 and 20 µL was selected for this study. It could be shown that this is a feasible volume for injection into the vitreous body of the eye from a marmoset. A transient effect to the intraocular pressure immediately after dosing was seen at 10 and 20 µL doses. However, this increased intraocular pressure did not result in any changes in the morphology of the eye that was evaluated microscopically. Therefore, both volumes could be used for future study. The electroretinogram showed that there were no changes during the study due to the intravitreal dosing of a placebo. In addition, a comparison to reference data from the cynomolgus monkeys (Table 2) shows that the cone-driven response appears to be stronger than in cynomolgus monkeys, which is likely to be related to the higher cone density found in marmosets.

## 5 Conclusion

It is concluded that, based on IOP measurements, ERG recording, and ophthalmologic and microscopic observations, up to 20 µL intravitreal injections of a placebo were well tolerated in the marmoset. Therefore, the common marmoset provides a potential alternative to the cynomolgus monkey for ocular toxicity testing.

## Supplement

10.5194/pb-4-93-2017-supplement The supplement related to this article is available online at: https://doi.org/10.5194/pb-4-93-2017-supplement.

## Data Availability

All measured ERG results are available in the Supplement.
