# Supplementary material for: Feasibility of intravitreal injections and ophthalmic safety assessment in marmoset (Callithrix jacchus) monkeys
Source: Primate Biol. 2017 Apr 28;4(1):93–100. doi: 10.5194/pb-4-93-2017 (PMC7041524; doi:10.5194/pb-4-93-2017)
Supplement: The supplement related to this article is available online at: https://doi.org/10.5194/pb-4-93-2017-supplement. [file pb-4-93-supplement.pdf]

Supplement of Primate Biol., 4, 93–100, 2017
http://www.primate-biol.net/4/93/2017/
doi:10.5194/pb-4-93-2017-supplement
© Author(s) 2017. CC Attribution 3.0 License.

Supplement of

Feasibility of intravitreal injections and ophthalmic safety assessment in marmoset (*Callithrix jacchus*) monkeys

Birgit Korbmacher et al.

Correspondence to: Birgit Korbmacher (birgit.korbmacher@covance.com, birgit.niggemann@covance.com)

The copyright of individual parts of the supplement might differ from the CC-BY 3.0 licence.

Table

Individual Electroretinogram

Test Item (dosage)	1	2
μL/both eyes bi-weekly (days 1, 15, 29 and 43)	10	20

Phase: Predose 1

Scotopic Measurement - b-Wave White Flash Rod Response - Amplitude (μV)

Group/ Sex	Animal Number	Left Eye 9.5 (mcds/m²)	Left Eye 30 (mcds/m²)	Left Eye 95 (mcds/m²)	Left Eye 300 (mcds/m²)	Left Eye 950 (mcds/m²)	Left Eye 3000 (mcds/m²)	Right Eye 9.5 (mcds/m²)	Right Eye 30 (mcds/m²)	Right Eye 95 (mcds/m²)	Right Eye 300 (mcds/m²)	Right Eye 950 (mcds/m²)	Right Eye 3000 (mcds/m²)
1/M	45366M	b	b	b	b	b	b	25.7	34.5	75.2	134.0	175.0	215.0
	45427M	b	b	b	b	b	b	42.0	44.6	102.0	134.0	206.0	184.0
	45504M	b	b	b	b	b	b	90.2	110.0	143.0	234.0	316.0	323.0
	Mean	-	-	-	-	-	-	52.6	63.0	106.7	167.3	232.3	240.7
	SD	-	-	-	-	-	-	33.5	41.0	34.1	57.7	74.1	73.0
	N	-	-	-	-	-	-	3	3	3	3	3	3
2/M	42737M	b	b	b	b	b	b	38.4	47.5	94.1	118.0	188.0	209.0
	45370M	b	b	b	b	b	b	48.8	73.6	76.8	135.0	163.0	218.0
	45500M	b	b	b	b	b	b	33.2	42.3	67.7	97.7	155.0	169.0
	Mean	-	-	-	-	-	-	40.1	54.5	79.5	116.9	168.7	198.7
	SD	-	-	-	-	-	-	7.9	16.8	13.4	18.7	17.2	26.1
	N	-	-	-	-	-	-	3	3	3	3	3	3

b = Not taken due to maximum sedation time was over

Table

Individual Electroretinogram

Test Item (dosage)	1	2
µL/both eyes bi-weekly (days 1, 15, 29 and 43)	10	20

Phase: Predose 2

Scotopic Measurement - b-Wave White Flash Rod Response - Amplitude (µV)

Group/ Sex	Animal Number	Left Eye 9.5 (mcds/m²)	Left Eye 30 (mcds/m²)	Left Eye 95 (mcds/m²)	Left Eye 300 (mcds/m²)	Left Eye 950 (mcds/m²)	Left Eye 3000 (mcds/m²)	Right Eye 9.5 (mcds/m²)	Right Eye 30 (mcds/m²)	Right Eye 95 (mcds/m²)	Right Eye 300 (mcds/m²)	Right Eye 950 (mcds/m²)	Right Eye 3000 (mcds/m²)
1/M	45366M	38.4	44.3	58.9	119.0	146.0	189.0	30.3	47.9	63.8	143.0	171.0	219.0
	45427M	32.9	39.4	58.3	75.8	130.0	143.0	37.1	26.0	66.4	87.6	179.0	198.0
	45504M	65.8	99.6	140.0	238.0	364.0	366.0	51.1	70.6	105.0	176.0	287.0	315.0
	Mean	45.7	61.1	85.7	144.3	213.3	232.7	39.5	48.2	78.4	135.5	212.3	244.0
	SD	17.6	33.4	47.0	84.0	130.7	117.7	10.6	22.3	23.1	44.7	64.8	62.4
	N	3	3	3	3	3	3	3	3	3	3	3	3
2/M	42737M	41.7	70.6	108.0	156.0	284.0	340.0	39.7	67.7	89.2	146.0	274.0	320.0
	45370M	34.8	21.8	30.3	92.4	153.0	178.0	23.1	29.6	36.1	63.8	90.5	131.0
	45500M	41.7	56.0	77.1	117.0	179.0	215.0	25.1	49.5	77.1	130.0	191.0	215.0
	Mean	39.4	49.5	71.8	121.8	205.3	244.3	29.3	48.9	67.5	113.3	185.2	222.0
	SD	4.0	25.0	39.1	32.1	69.4	84.9	9.1	19.1	27.8	43.6	91.9	94.7
	N	3	3	3	3	3	3	3	3	3	3	3	3

Table

Individual Electroretinogram

Test Item (dosage)	1	2
µL/both eyes bi-weekly (days 1, 15, 29 and 43)	10	20

Phase		Dosing Week 6											
		Scotopic Measurement - b-Wave White Flash Rod Response - Amplitude (µV)											
		Left Eye						Right Eye					
Group/Sex	Animal Number	9.5 (mcds/m^2)	30 (mcds/m^2)	95 (mcds/m^2)	300 (mcds/m^2)	950 (mcds/m^2)	3000 (mcds/m^2)	9.5 (mcds/m^2)	30 (mcds/m^2)	95 (mcds/m^2)	300 (mcds/m^2)	950 (mcds/m^2)	3000 (mcds/m^2)
1/M	45366M	19.9	27.3	52.7	103.0	142.0	176.0	35.5	34.2	54.7	127.0	182.0	218.0
	45427M	37.1	78.1	71.0	148.0	186.0	199.0	34.5	78.8	73.2	139.0	198.0	211.0
	45504M	20.2	30.3	48.8	92.1	151.0	201.0	24.7	51.4	88.9	181.0	233.0	267.0
	Mean	25.7	45.2	57.5	114.4	159.7	192.0	31.6	54.8	72.3	149.0	204.3	232.0
	SD	9.8	28.5	11.9	29.6	23.2	13.9	6.0	22.5	17.1	28.4	26.1	30.5
	N	3	3	3	3	3	3	3	3	3	3	3	3
2/M	42737M	45.9	53.1	93.1	154.0	244.0	288.0	41.0	53.4	87.2	168.0	253.0	310.0
	45370M	24.7	10.1	42.3	117.0	162.0	210.0	27.7	17.6	80.7	96.4	142.0	208.0
	45500M	63.5	45.9	63.8	85.6	177.0	223.0	29.0	15.0	10.7	45.6	103.0	113.0
	Mean	44.7	36.4	66.4	118.9	194.3	240.3	32.6	28.7	59.5	103.3	166.0	210.3
	SD	19.4	23.0	25.5	34.2	43.7	41.8	7.3	21.5	42.4	61.5	77.8	98.5
	N	3	3	3	3	3	3	3	3	3	3	3	3

Table

Individual Electroretinogram

Test Item (dosage)	1	2
µL/both eyes bi-weekly (days 1, 15, 29 and 43)	10	20

Phase		Predose 1											
		Scotopic Measurement - b-Wave White Flash Rod Response - Peak Latency (ms)											
		Left Eye						Right Eye					
Group/Sex	Animal Number	9.5 (mcds/m²)	30 (mcds/m²)	95 (mcds/m²)	300 (mcds/m²)	950 (mcds/m²)	3000 (mcds/m²)	9.5 (mcds/m²)	30 (mcds/m²)	95 (mcds/m²)	300 (mcds/m²)	950 (mcds/m²)	3000 (mcds/m²)
1/M	45366M	b	b	b	b	b	b	70.3	55.5	34.3	32.8	30.5	29.8
	45427M	b	b	b	b	b	b	57.3	44.3	34.3	32.8	33.0	31.5
	45504M	b	b	b	b	b	b	54.0	41.0	38.8	33.8	34.3	35.3
	Mean	-	-	-	-	-	-	60.5	46.9	35.8	33.1	32.6	32.2
	SD	-	-	-	-	-	-	8.6	7.6	2.6	0.6	1.9	2.8
	N	-	-	-	-	-	-	3	3	3	3	3	3
2/M	42737M	b	b	b	b	b	b	56.5	47.5	35.5	33.0	33.5	34.3
	45370M	b	b	b	b	b	b	79.0	59.5	38.8	32.3	30.5	30.3
	45500M	b	b	b	b	b	b	64.3	49.3	40.5	35.8	35.0	35.8
	Mean	-	-	-	-	-	-	66.6	52.1	38.3	33.7	33.0	33.5
	SD	-	-	-	-	-	-	11.4	6.5	2.5	1.9	2.3	2.8
	N	-	-	-	-	-	-	3	3	3	3	3	3

b = Not taken due to maximum sedation time was over

Table

Individual Electroretinogram

Test Item (dosage)	1	2
µL/both eyes bi-weekly (days 1, 15, 29 and 43)	10	20

Phase		Predose 2											
		Scotopic Measurement - b-Wave White Flash Rod Response - Peak Latency (ms)											
		Left Eye						Right Eye					
Group/Sex	Animal Number	9.5 (mcds/m^2)	30 (mcds/m^2)	95 (mcds/m^2)	300 (mcds/m^2)	950 (mcds/m^2)	3000 (mcds/m^2)	9.5 (mcds/m^2)	30 (mcds/m^2)	95 (mcds/m^2)	300 (mcds/m^2)	950 (mcds/m^2)	3000 (mcds/m^2)
1/M	45366M	69.0	61.0	43.5	35.3	33.5	32.0	59.8	55.8	40.3	34.3	32.0	31.3
	45427M	60.0	48.5	37.8	35.3	35.5	35.3	61.5	56.8	43.8	34.5	35.0	35.5
	45504M	50.5	43.5	38.0	36.5	36.5	36.5	50.3	44.5	37.8	36.3	35.8	36.0
	Mean	59.8	51.0	39.8	35.7	35.2	34.6	57.2	52.4	40.6	35.0	34.3	34.3
	SD	9.3	9.0	3.2	0.7	1.5	2.3	6.0	6.8	3.0	1.1	2.0	2.6
	N	3	3	3	3	3	3	3	3	3	3	3	3
2/M	42737M	71.5	60.8	41.8	33.0	32.8	34.0	72.5	61.3	47.8	33.5	32.5	33.5
	45370M	92.3	71.3	41.0	40.0	40.0	40.3	84.8	67.0	47.0	39.0	39.0	39.0
	45500M	74.8	52.5	42.8	34.8	36.0	36.5	70.3	53.5	42.8	35.0	36.0	37.0
	Mean	79.5	61.5	41.9	35.9	36.3	36.9	75.9	60.6	45.9	35.8	35.8	36.5
	SD	11.2	9.4	0.9	3.6	3.6	3.2	7.8	6.8	2.7	2.8	3.3	2.8
	N	3	3	3	3	3	3	3	3	3	3	3	3

Table

Individual Electroretinogram

Test Item (dosage)	1	2
µL/both eyes bi-weekly (days 1, 15, 29 and 43)	10	20

Phase: Dosing Week 6

Scotopic Measurement - b-Wave White Flash Rod Response - Peak Latency (ms)

Group/ Sex	Animal Number	Left Eye 9.5 ($mcds/m^2$)	Left Eye 30 ($mcds/m^2$)	Left Eye 95 ($mcds/m^2$)	Left Eye 300 ($mcds/m^2$)	Left Eye 950 ($mcds/m^2$)	Left Eye 3000 ($mcds/m^2$)	Right Eye 9.5 ($mcds/m^2$)	Right Eye 30 ($mcds/m^2$)	Right Eye 95 ($mcds/m^2$)	Right Eye 300 ($mcds/m^2$)	Right Eye 950 ($mcds/m^2$)	Right Eye 3000 ($mcds/m^2$)
1/M	45366M	70.8	59.8	38.3	34.3	34.5	32.8	67.8	59.0	34.3	33.3	33.3	33.0
	45427M	58.5	48.5	34.8	33.5	33.5	32.0	55.8	47.5	36.0	33.0	33.0	31.3
	45504M	71.5	51.3	46.3	33.8	32.3	30.8	64.8	47.8	33.5	32.8	30.8	30.8
	Mean	66.9	53.2	39.8	33.9	33.4	31.9	62.8	51.4	34.6	33.0	32.4	31.7
	SD	7.3	5.9	5.9	0.4	1.1	1.0	6.2	6.6	1.3	0.3	1.4	1.2
	N	3	3	3	3	3	3	3	3	3	3	3	3
2/M	42737M	67.8	60.0	46.5	33.0	34.5	35.3	66.0	56.5	46.5	32.8	34.5	35.3
	45370M	66.3	51.3	36.8	36.3	35.5	34.0	65.3	58.3	33.0	32.8	33.0	33.8
	45500M	73.0	56.8	44.5	38.0	37.8	38.0	69.3	59.0	45.8	37.5	37.5	37.5
	Mean	69.0	56.0	42.6	35.8	35.9	35.8	66.9	57.9	41.8	34.4	35.0	35.5
	SD	3.5	4.4	5.1	2.5	1.7	2.0	2.1	1.3	7.6	2.7	2.3	1.9
	N	3	3	3	3	3	3	3	3	3	3	3	3

Table

Individual Electroretinogram

Test Item (dosage)	1	2
µL/both eyes bi-weekly (days 1, 15, 29 and 43)	10	20

Group/ Sex	Animal Number	Predose 1				Predose 2			
		Scotopic Measurement - a-Wave White Flash Rod Response (3000 mcds/m^2)							
		Amplitude (µV)		Peak Latency (ms)		Amplitude (µV)		Peak Latency (ms)	
		Left Eye	Right Eye	Left Eye	Right Eye	Left Eye	Right Eye	Left Eye	Right Eye
1/M	45366M	b	121.0	b	13.5	98.6	118.0	14.5	14.3
	45427M	b	132.0	b	13.8	117.0	118.0	16.0	15.0
	45504M	b	177.0	b	13.5	180.0	134.0	14.5	14.5
	Mean	-	143.3	-	13.6	131.9	123.3	15.0	14.6
	SD	-	29.7	-	0.2	42.7	9.2	0.9	0.4
	N	-	3	-	3	3	3	3	3
2/M	42737M	b	123.0	b	13.8	139.0	128.0	14.0	13.8
	45370M	b	199.0	b	14.0	148.0	114.0	16.3	19.5
	45500M	b	104.0	b	14.8	118.0	122.0	14.5	14.8
	Mean	-	142.0	-	14.2	135.0	121.3	14.9	16.0
	SD	-	50.3	-	0.5	15.4	7.0	1.2	3.0
	N	-	3	-	3	3	3	3	3

b = Not taken due to maximum sedation time was over

Table

Individual Electroretinogram

Test Item (dosage)	1	2
µL/both eyes bi-weekly (days 1, 15, 29 and 43)	10	20

	Phase	Dosing Week 6			
		Scotopic Measurement - a-Wave White Flash Rod Response (3000 mcds/m^2)			
Group/	Animal	Amplitude (µV)		Peak Latency (ms)	
Sex	Number	Left Eye	Right Eye	Left Eye	Right Eye
1/M	45366M	93.8	117.0	15.3	15.0
	45427M	159.0	149.0	14.3	14.0
	45504M	60.2	124.0	15.0	14.3
	Mean	104.3	130.0	14.9	14.4
	SD	50.2	16.8	0.5	0.5
	N	3	3	3	3
2/M	42737M	157.0	170.0	14.3	14.5
	45370M	180.0	178.0	16.3	16.0
	45500M	119.0	66.1	15.5	15.5
	Mean	152.0	138.0	15.4	15.3
	SD	30.8	62.4	1.0	0.8
	N	3	3	3	3

Table

Individual Electroretinogram

Test Item (dosage)	1	2
µL/both eyes bi-weekly (days 1, 15, 29 and 43)	10	20

Group/ Sex	Animal Number	Phase: Predose 1				Predose 2			
		Scotopic Measurement - Oscillatory Potentials (3000 mcds/m^2)							
		Amplitude (µV)		Peak Latency (ms)		Amplitude (µV)		Peak Latency (ms)	
		Left Eye	Right Eye	Left Eye	Right Eye	Left Eye	Right Eye	Left Eye	Right Eye
1/M	45366M	b	12.3	b	22.0	4.6	9.8	22.8	22.9
	45427M	b	23.4	b	22.2	9.6	9.3	26.3	24.7
	45504M	b	39.7	b	22.9	25.2	28.0	24.1	24.3
	Mean	-	25.1	-	22.4	13.1	15.7	24.4	24.0
	SD	-	13.8	-	0.5	10.8	10.6	1.8	0.9
	N	-	3	-	3	3	3	3	3
2/M	42737M	b	13.9	b	22.7	24.3	21.6	22.5	22.3
	45370M	b	18.7	b	22.7	16.6	17.2	20.8	23.6
	45500M	b	11.8	b	27.5	8.8	7.9	23.9	24.0
	Mean	-	14.8	-	24.3	16.6	15.6	22.4	23.3
	SD	-	3.5	-	2.8	7.7	7.0	1.6	0.9
	N	-	3	-	3	3	3	3	3

b = Not taken due to maximum sedation time was over

Table

Individual Electroretinogram

Test Item (dosage)	1	2
μL/both eyes bi-weekly (days 1, 15, 29 and 43)	10	20

Group/ Sex	Animal Number	Phase: Dosing Week 6 — Scotopic Measurement - Oscillatory Potentials (3000 mcds/m^2) Amplitude (μV) Left Eye	Amplitude (μV) Right Eye	Peak Latency (ms) Left Eye	Peak Latency (ms) Right Eye
1/M	45366M	9.0	12.8	27.7	28.7
	45427M	15.2	13.3	22.9	23.0
	45504M	13.7	28.9	26.4	25.3
	Mean	12.6	18.3	25.7	25.7
	SD	3.2	9.2	2.5	2.9
	N	3	3	3	3
2/M	42737M	22.9	24.7	25.5	25.1
	45370M	23.4	16.0	29.1	28.6
	45500M	14.1	8.4	27.8	26.8
	Mean	20.1	16.4	27.5	26.8
	SD	5.2	8.2	1.8	1.8
	N	3	3	3	3

Table

Individual Electroretinogram

Test Item (dosage)	1	2
μL/both eyes bi-weekly (days 1, 15, 29 and 43)	10	20

Group/ Sex	Animal Number	Predose 1				Predose 2			
		Photopic Measurement - Flicker (30 Hz) - Amplitude (μV)							
		0 min		10 min		0 min		10 min	
		Left Eye	Right Eye	Left Eye	Right Eye	Left Eye	Right Eye	Left Eye	Right Eye
1/M	45366M	b	118.0	b	167.0	93.3	112.0	107.0	146.0
	45427M	b	109.0	b	155.0	68.2	109.0	119.0	149.0
	45504M	b	183.0	b	223.0	190.0	151.0	176.0	140.0
	Mean	-	136.7	-	181.7	117.2	124.0	134.0	145.0
	SD	-	40.4	-	36.3	64.3	23.4	36.9	4.6
	N	-	3	-	3	3	3	3	3
2/M	42737M	b	134.0	b	172.0	205.0	193.0	238.0	219.0
	45370M	b	134.0	b	192.0	111.0	75.4	122.0	86.4
	45500M	b	97.0	b	110.0	113.0	113.0	88.1	115.0
	Mean	-	121.7	-	158.0	143.0	127.1	149.4	140.1
	SD	-	21.4	-	42.8	53.7	60.1	78.6	69.8
	N	-	3	-	3	3	3	3	3

b = Not taken due to maximum sedation time was over

Table

Individual Electroretinogram

Test Item (dosage)	1	2
μL/both eyes bi-weekly (days 1, 15, 29 and 43)	10	20

		Dosing Week 6			
		Photopic Measurement - Flicker (30 Hz) - Amplitude (μV)			
Group/	Animal	0 min		10 min	
Sex	Number	Left Eye	Right Eye	Left Eye	Right Eye
1/M	45366M	72.3	98.3	97.8	135.0
	45427M	118.0	126.0	158.0	173.0
	45504M	91.6	139.0	207.0	181.0
	Mean	94.0	121.1	154.3	163.0
	SD	22.9	20.8	54.7	24.6
	N	3	3	3	3
2/M	42737M	156.0	169.0	186.0	225.0
	45370M	111.0	104.0	120.0	123.0
	45500M	95.1	58.1	75.8	50.0
	Mean	120.7	110.4	127.3	132.7
	SD	31.6	55.7	55.5	87.9
	N	3	3	3	3

Table

Individual Electroretinogram

Test Item (dosage)	1	2
µL/both eyes bi-weekly (days 1, 15, 29 and 43)	10	20

	Phase	Predose 1				Predose 2			
		Photopic Measurement - Flicker (30 Hz) - Peak Latency (ms)							
Group/	Animal	0 min		10 min		0 min		10 min	
Sex	Number	Left Eye	Right Eye	Left Eye	Right Eye	Left Eye	Right Eye	Left Eye	Right Eye
1/M	45366M	-	56.4	-	57.0	57.4	57.4	57.4	57.4
	45427M	-	57.0	-	58.8	59.0	58.0	58.0	58.0
	45504M	-	57.4	-	57.4	57.8	58.0	58.0	58.4
	Mean	-	56.9	-	57.7	58.1	57.8	57.8	57.9
	SD	-	0.5	-	0.9	0.8	0.3	0.3	0.5
	N	-	3	-	3	3	3	3	3
2/M	42737M	-	58.2	-	57.6	56.6	56.4	56.4	56.2
	45370M	-	57.4	-	57.4	59.6	60.0	60.0	59.4
	45500M	-	57.8	-	58.4	58.0	58.0	58.0	58.0
	Mean	-	57.8	-	57.8	58.1	58.1	58.1	57.9
	SD	-	0.4	-	0.5	1.5	1.8	1.8	1.6
	N	-	3	-	3	3	3	3	3

Table

Individual Electroretinogram

Test Item (dosage)	1	2
µL/both eyes bi-weekly (days 1, 15, 29 and 43)	10	20

		Dosing Week 6			
		Photopic Measurement - Flicker (30 Hz) - Peak Latency (ms)			
		0 min		10 min	
Group/ Sex	Animal Number	Left Eye	Right Eye	Left Eye	Right Eye
1/M	45366M	58.6	58.6	59.0	59.0
	45427M	58.2	58.2	58.0	58.0
	45504M	58.2	58.6	57.8	57.6
	Mean	58.3	58.5	58.3	58.2
	SD	0.2	0.2	0.6	0.7
	N	3	3	3	3
2/M	42737M	57.6	57.6	57.2	56.8
	45370M	60.0	59.8	60.6	60.8
	45500M	59.4	59.4	59.8	59.4
	Mean	59.0	58.9	59.2	59.0
	SD	1.2	1.2	1.8	2.0
	N	3	3	3	3

Table

Individual Electroretinogram

Test Item (dosage)	1	2
µL/both eyes bi-weekly (days 1, 15, 29 and 43)	10	20

	Phase	Predose 1				Predose 2			
		Photopic Measurement - b-Wave Red Flash Cone Response - Amplitude (µV)							
		Left Eye		Right Eye		Left Eye		Right Eye	
Group/ Sex	Animal Number	300 (mcds/m^2)	950	300 (mcds/m^2)	950	300 (mcds/m^2)	950	300 (mcds/m^2)	950
1/M	45366M	b	b	49.0	110.0	22.9	86.3	35.0	111.0
	45427M	b	b	74.5	101.0	42.5	91.1	44.6	115.0
	45504M	b	b	110.0	173.0	53.5	172.0	61.4	121.0
	Mean	-	-	77.8	128.0	39.6	116.5	47.0	115.7
	SD	-	-	30.6	39.2	15.5	48.2	13.4	5.0
	N	-	-	3	3	3	3	3	3
2/M	42737M	b	b	37.8	110.0	38.2	143.0	31.4	125.0
	45370M	b	b	60.5	123.0	28.0	113.0	13.7	86.9
	45500M	b	b	23.8	82.7	16.3	73.9	24.4	91.0
	Mean	-	-	40.7	105.2	27.5	110.0	23.2	101.0
	SD	-	-	18.5	20.6	11.0	34.6	8.9	20.9
	N	-	-	3	3	3	3	3	3

b = Not taken due to maximum sedation time was over

Table

Individual Electroretinogram

Test Item (dosage)	1	2
µL/both eyes bi-weekly (days 1, 15, 29 and 43)	10	20

Phase		Dosing Week 6			
		Photopic Measurement - b-Wave Red Flash Cone Response - Amplitude (µV)			
		Left Eye		Right Eye	
Group/ Sex	Animal Number	300 (mcds/m^2)	950 (mcds/m^2)	300 (mcds/m^2)	950 (mcds/m^2)
1/M	45366M	35.8	75.4	45.1	108.0
	45427M	67.7	113.0	68.4	131.0
	45504M	99.4	143.0	85.6	127.0
	Mean	67.6	110.5	66.4	122.0
	SD	31.8	33.9	20.3	12.3
	N	3	3	3	3
2/M	42737M	37.4	123.0	39.2	139.0
	45370M	35.3	108.0	40.5	104.0
	45500M	16.1	58.4	14.3	50.5
	Mean	29.6	96.5	31.3	97.8
	SD	11.7	33.8	14.8	44.6
	N	3	3	3	3

Table

Individual Electroretinogram

Test Item (dosage)	1	2
µL/both eyes bi-weekly (days 1, 15, 29 and 43)	10	20

Phase		Predose 1				Predose 2			
		Photopic Measurement - b-Wave Red Flash Cone Response - Peak Latency (ms)							
		Left Eye		Right Eye		Left Eye		Right Eye	
Group/ Sex	Animal Number	300 (mcds/m^2)	950 (mcds/m^2)	300 (mcds/m^2)	950 (mcds/m^2)	300 (mcds/m^2)	950 (mcds/m^2)	300 (mcds/m^2)	950 (mcds/m^2)
1/M	45366M	-	-	22.2	20.9	23.2	22.0	23.3	21.7
	45427M	-	-	22.6	23.4	23.9	23.0	23.7	22.7
	45504M	-	-	22.2	21.2	24.7	22.5	23.8	22.4
	Mean	-	-	22.3	21.8	23.9	22.5	23.6	22.3
	SD	-	-	0.2	1.4	0.8	0.5	0.3	0.5
	N	-	-	3	3	3	3	3	3
2/M	42737M	-	-	22.5	21.1	21.1	20.5	21.0	20.5
	45370M	-	-	23.2	21.3	28.5	25.0	27.1	26.0
	45500M	-	-	25.8	22.7	24.9	23.3	25.4	23.1
	Mean	-	-	23.8	21.7	24.8	22.9	24.5	23.2
	SD	-	-	1.7	0.9	3.7	2.3	3.1	2.8
	N	-	-	3	3	3	3	3	3

Table

Individual Electroretinogram

Test Item (dosage)	1	2
μL/both eyes bi-weekly (days 1, 15, 29 and 43)	10	20

Group/Sex	Animal Number	Dosing Week 6: Photopic Measurement - b-Wave Red Flash Cone Response - Peak Latency (ms)			
		Left Eye		Right Eye	
		300 (mcds/m^2)	950 (mcds/m^2)	300 (mcds/m^2)	950 (mcds/m^2)
1/M	45366M	24.1	22.9	24.7	23.5
	45427M	22.2	22.4	22.9	22.3
	45504M	21.3	21.9	22.2	22.2
	Mean	22.5	22.4	23.3	22.7
	SD	1.4	0.5	1.3	0.7
	N	3	3	3	3
2/M	42737M	21.7	21.5	21.4	21.2
	45370M	28.5	25.6	27.5	25.2
	45500M	27.8	27.8	24.3	25.0
	Mean	26.0	25.0	24.4	23.8
	SD	3.7	3.2	3.1	2.3
	N	3	3	3	3

Table

Individual Electroretinogram

Test Item (dosage)	1	2
µL/both eyes bi-weekly (days 1, 15, 29 and 43)	10	20

Group/ Sex	Animal Number	Predose 1 Amplitude (µV) Left Eye	Predose 1 Amplitude (µV) Right Eye	Predose 1 Peak Latency (ms) Left Eye	Predose 1 Peak Latency (ms) Right Eye	Predose 2 Amplitude (µV) Left Eye	Predose 2 Amplitude (µV) Right Eye	Predose 2 Peak Latency (ms) Left Eye	Predose 2 Peak Latency (ms) Right Eye
		Photopic Measurement - b-Wave White Flash Cone Response (3000 mcds/m^2)							
1/M	45366M	b	179.0	b	25.9	125.0	163.0	25.4	25.4
	45427M	b	193.0	b	27.5	143.0	171.0	26.3	26.1
	45504M	b	295.0	b	25.3	223.0	196.0	26.8	26.8
	Mean	-	222.3	-	26.2	163.7	176.7	26.2	26.1
	SD	-	63.3	-	1.1	52.2	17.2	0.7	0.7
	N	-	3	-	3	3	3	3	3
2/M	42737M	b	210.0	b	25.1	282.0	258.0	23.4	23.1
	45370M	b	221.0	b	25.3	a	a	a	a
	45500M	b	144.0	b	26.7	123.0	145.0	25.1	25.1
	Mean	-	191.7	-	25.7	202.5	201.5	24.3	24.1
	SD	-	41.6	-	0.9	112.4	79.9	1.2	1.4
	N	-	3	-	3	2	2	2	2

a = No measurement

b = Not taken due to maximum sedation time was over

Table

Individual Electroretinogram

Test Item (dosage)	1	2
µL/both eyes bi-weekly (days 1, 15, 29 and 43)	10	20

Group/ Sex	Animal Number	Dosing Week 6: Photopic Measurement - b-Wave White Flash Cone Response (3000 mcds/m^2) Amplitude (µV) Left Eye	Amplitude (µV) Right Eye	Peak Latency (ms) Left Eye	Peak Latency (ms) Right Eye
1/M	45366M	121.0	169.0	27.2	27.7
	45427M	200.0	223.0	26.1	26.1
	45504M	286.0	234.0	25.8	26.2
	Mean	202.3	208.7	26.4	26.7
	SD	82.5	34.8	0.7	0.9
	N	3	3	3	3
2/M	42737M	242.0	279.0	24.5	24.0
	45370M	163.0	166.0	30.4	30.3
	45500M	132.0	124.0	28.5	28.3
	Mean	179.0	189.7	27.8	27.5
	SD	56.7	80.2	3.0	3.2
	N	3	3	3	3

Table

Individual Electroretinogram

Test Item (dosage)	1	2
μL/both eyes bi-weekly (days 1, 15, 29 and 43)	10	20

	Phase	Predose 1				Predose 2			
		Photopic Measurement - a-Wave White Flash Cone Response (3000 mcds/m^2)							
Group/	Animal	Amplitude (μV)		Peak Latency (ms)		Amplitude (μV)		Peak Latency (ms)	
Sex	Number	Left Eye	Right Eye	Left Eye	Right Eye	Left Eye	Right Eye	Left Eye	Right Eye
1/M	45366M	b	35.0	b	14.8	29.5	36.3	14.8	14.8
	45427M	b	42.6	b	14.4	45.2	52.6	15.7	15.5
	45504M	b	48.3	b	14.2	34.0	55.2	15.9	15.2
	Mean	-	42.0	-	14.5	36.2	48.0	15.5	15.2
	SD	-	6.7	-	0.3	8.1	10.2	0.6	0.4
	N	-	3	-	3	3	3	3	3
2/M	42737M	b	41.2	b	14.3	58.1	52.2	14.2	14.2
	45370M	b	57.6	b	14.6	a	a	a	a
	45500M	b	37.8	b	14.8	37.1	44.8	15.6	15.6
	Mean	-	45.5	-	14.6	47.6	48.5	14.9	14.9
	SD	-	10.6	-	0.3	14.8	5.2	1.0	1.0
	N	-	3	-	3	2	2	2	2

a = No measurement

b = Not taken due to maximum sedation time was over

Table

Individual Electroretinogram

Test Item (dosage)	1	2
μL/both eyes bi-weekly (days 1, 15, 29 and 43)	10	20

Group/ Sex	Animal Number	Dosing Week 6			
		Photopic Measurement - a-Wave White Flash Cone Response (3000 mcds/m^2)			
		Amplitude (μV)		Peak Latency (ms)	
		Left Eye	Right Eye	Left Eye	Right Eye
1/M	45366M	26.0	36.5	15.4	15.9
	45427M	47.0	54.2	14.5	15.0
	45504M	41.5	35.8	14.1	14.1
	Mean	38.2	42.2	14.7	15.0
	SD	10.9	10.4	0.7	0.9
	N	3	3	3	3
2/M	42737M	50.9	56.6	14.8	14.6
	45370M	57.5	52.2	17.4	17.3
	45500M	31.6	25.7	15.9	16.1
	Mean	46.7	44.8	16.0	16.0
	SD	13.5	16.7	1.3	1.4
	N	3	3	3	3
